# Sensing Based on Fano-Type Resonance Response of All-Dielectric Metamaterials

**DOI:** 10.3390/s150409344

**Published:** 2015-04-21

**Authors:** Elena Semouchkina, Ran Duan, George Semouchkin, Ravindra Pandey

**Affiliations:** 1 Department of Electrical and Computer Engineering, Michigan Technological University, 1400 Townsend Drive, Houghton, MI 49931, USA; E-Mail: gbsemouc@mtu.edu; 2 Department of Physics, Michigan Technological University, 1400 Townsend Drive, Houghton, MI 49931, USA; E-Mails: rduan@mtu.edu (R.D.); pandey@mtu.edu (R.P.)

**Keywords:** Fano resonance, dielectric resonator, metamaterial

## Abstract

A new sensing approach utilizing Mie resonances in metamaterial arrays composed of dielectric resonators is proposed. These arrays were found to exhibit specific, extremely high-Q factor (up to 15,000) resonances at frequencies corresponding to the lower edge of the array second transmission band. The observed resonances possessed with features typical for Fano resonances (FRs), which were initially revealed in atomic processes and recently detected in macro-structures, where they resulted from interference between local resonances and a continuum of background waves. Our studies demonstrate that frequencies and strength of Fano-type resonances in all-dielectric arrays are defined by interaction between local Mie resonances and Fabry-Perot oscillations of Bloch eigenmodes that makes possible controlling the resonance responses by changing array arrangements. The opportunity for obtaining high-Q responses in compact arrays is investigated and promising designs for sensing the dielectric properties of analytes in the ambient are proposed.

## 1. Introduction

Until recently, the phenomenon of Fano resonance (FR) has been attributed to a special type of electron scattering in atomic systems. In the last decade FRs have been also revealed in planar periodic structures, such as slabs of photonic crystals (PhCs), 2D metamaterials (MMs) and plasmonic structures systems, however, there have been only a few reports about FR observations at microwave and mm-wave frequencies and only in structures of specifically combined resonators with broken symmetry.

Although FRs demonstrate a peculiar type of line shape, they can originate from conventional resonances of Lorentz-Mie type under special conditions provided by interference between locally initiated scattered waves and a continuum of background modes formed in multi-resonator systems under wave incidence [1,2,3]. In difference from the latter “bright” modes, the former “dark” modes are weakly coupled to incident waves, however, they can be pumped due to interaction with “bright” modes. Constructive interference can make the resonance response much stronger in a very narrow frequency band thus increasing the Q-factor of the resonance up to very high values. It was also noted that FRs can be associated with a coherent response of multi-resonator systems and with lossless slow wave transmission through the host medium *i.e.*, the phenomenon comparable to Electromagnetically Induced Transparency (EIT) known in atomic physics [1,4]. 

The possibility to control the line-shape of FRs and to provide very high Q-factors of these resonances opens up exciting opportunities for advancing the science of resonance sensing. Since the efficiency of resonance sensing depends on the Q-factor of the employed resonance, low-loss dielectric resonators (DRs) represent desirable components of resonance sensors. However, typical Q factors for isolated DRs exhibiting Mie/Lorentz-type resonance rarely exceed 150. Therefore, even though DR-based microwave sensors are widely used for detecting the presence of various liquids and gases in the ambient media and in customized solutions, the sensitivity of these sensors is far not sufficient for advanced applications, especially in biochemistry and biomedicine [5,6,7,8]. The use of FRs, which potentially could achieve Q factors up to 5000–15,000, promises to enhance the resonance sensing efficiency significantly. The works on plasmonic MMs [1,2,9] as well as on meta-surfaces [10,11,12], in particular, on those formed of DRs [13] have already proved new opportunities for sensing based on FRs in THz and optical range. Comparably high-Q values have been achieved only at resonances of Whispering Gallery Modes (WGM) [14] or in resonance cavities including built-in PhC defects of periodicity [15], however, excitation of these resonances demanded more complicated designs and fabrication techniques compared to metasurfaces. In addition, it is challenging in practice to vary Q-factors of these resonances, while Q-factor control is often required for matching the parameters of the resonance and the sensitivity of peak detectors used for resonance surveillance.

In this work we propose a new MM-based sensing approach for microwave testing. The proposed sensors utilize high-Q resonances [16] that produce unified (integrated) resonance response in 3D all-dielectric MMs formed from identical ceramic cylindrical DRs, which are often used in microwave antennas, filters, wave-guiding systems and ordinary sensors. We investigate the origin of high-Q resonances in all-dielectric MMs and demonstrate the possibility to obtain responses with the desired specifics in compact resonance structures. We present an example of a MM structure, which could be used for sensing, and investigate its efficiency for sensing the dielectric permittivity of an analyte introduced in the ambient. Full-wave simulations were performed by using CST Microwave Studio and COMSOL Multiphysics software packages. Band diagrams of the arrays were simulated by using the MPB software developed for photonic crystal studies [17].

## 2. Specifics of FRs and Their Appearance in PhCs and Planar MMs

Resonances are known to be a universal characteristic of many types of classical and quantum systems. The spectral dependence (line shape) of elementary resonances including the Mie resonance [18] is usually described by the well-known Lorentzian function [19], which predicts a symmetric bell-shape response:

(1) $$I\left(\omega \right)=\frac{\gamma^{2}}{\gamma^{2}+{\left(\omega -\omega_{res}\right)}^{2}}$$

where *I* is the normalized magnitude of resonance oscillations, *ω* is driving frequency, and *ω_res_* and *γ* are the spectral position and the half-width of the resonance response (at the 3 dB level).

In the 60 s a new type of resonance called later FR was revealed in the studies of inelastic electron scattering from helium atoms [20,21]. Scattering process was complicated by autoionization of helium atoms with subsequent spontaneous deionization accompanied by ejecting of electrons. It was shown that interference between simple scattering (without ionization) and scattering via autoionization could create asymmetry in the scattering line shape with the line width close to the inverse autoionization time. Employing the concept of interference between broadband and resonance-type responses allows for describing a variety of asymmetric line shapes by the function:

(2) $$I*\left(\omega \right)=\frac{{\left(q\gamma +\omega -\omega_{res}\right)}^{2}}{\gamma^{2}+{\left(\omega -\omega_{res}\right)}^{2}}$$

where *q* is the Fano parameter characterizing the degree of asymmetry. The formula suggests the presence of one minimum (to one side of *ω_res_*) and one maximum (to the other side of *ω_res_*) in the Fano profile due to, respectively, destructive or constructive interference between components of responses. The switch in the character of interference can be related to changing the phase of resonance scattering by *π* at the resonance frequency.

**Figure 1:**
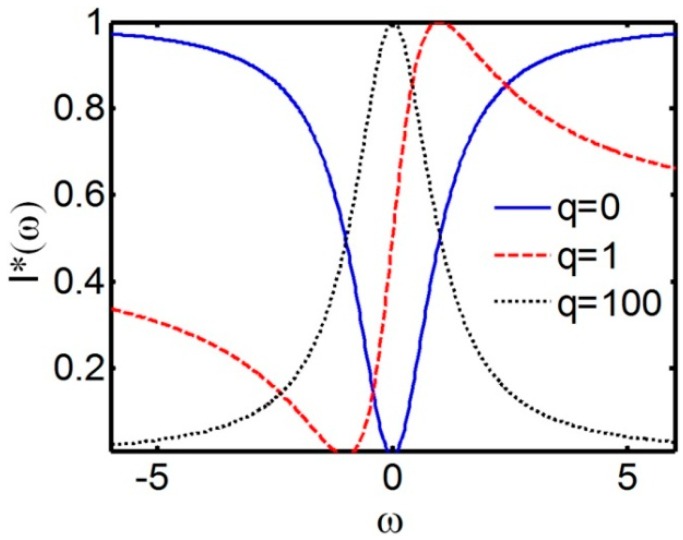
Normalized Fano resonance profiles for various values of the asymmetry parameter *q* versus normalized frequency, here I* is the normalized resonance amplitude and γ is the full width at half maximum of I*.

As seen from Figure 1, at |*q*| → ∞ asymmetry disappears and the Lorentz profile is observed, while at *q* = 0 the anti-resonance defines the line shape. Typical asymmetrical Fano profile is seen at *q* = 1, when maximal and zero scattering are shifted in opposite directions with respect to the resonance frequency by 1/*q* and *q*, respectively.

Following the discovery of FRs in [20,21], they were detected in many structures, such as quantum dots, nanowires, and tunnel junctions. However, until the beginning of this century, all observed FRs were regarded as phenomena entirely specific to quantum systems. First observations of FRs in macro-systems were done in planar dielectric slabs with periodically arranged air holes, where FRs looked as asymmetric singularities in scattering spectra [22,23]. It was suggested in [23,24] that the character of the slab response was defined by interference between the incident wave continuum characterized by relatively weak dependence on frequency/time and so-called guided resonances (GRs) positioned in the PhC band diagram near transmission bands edges. FRs of a different origin were detected in specifically disordered synthetic opals with liquid fillers [25]. The phenomenon was explained by interference between the narrow band Bragg and the dispersed Mie scattering from SiO_2_ particles, which were neither perfectly spherical, nor uniform in size or homogeneous in dielectric permittivity. 

Fano resonances in 2D MMs were first detected in structures composed of split rings representing two wire arcs of different lengths [26]. At normal wave incidence on the structure, its transmission spectrum was found to demonstrate a transmission peak between two deep drops. This peak was related by the authors to the dark resonance mode defined by formation in SRRs magnetic moments normal to the array plane, while the drops were related to two slightly different bright resonance modes supported by in-plane oscillations of currents in SRR wire branches. According to Papasimakis *et al*. [4], interference between two bright modes could suppress scattering and cause EIT at the frequency of the dark mode. Similar transparency was observed earlier in [27].

Following [4,26], various planar (2D) MMs with complicated constituents supporting several overlapping resonances [28,29,30,31,32,33] were designed to obtain either FRs or the resonance related EIT, which was found to occur at wave group velocities close to zero, *i.e.*, at slow waves. It is worth mentioning here that sharp FR-related transmission peaks were first observed in perforated dielectric slabs with simple symmetric unit cells [23], while in a later work on stacked fishnets formed from perforated metal films sharp FRs and EIT were obtained only when regular elliptic apertures in films were replaced by dimers of half-ellipses to cause the Woods anomaly [34]. The authors of [35] confirmed the result of [23] and showed that symmetry-breaking in unit cells was not necessary for realizing FRs and EIT, however, the Q factor of resonances that they obtained in symmetric cells did not exceed 40. Meanwhile later, as mentioned above, it was shown that the FR-like resonances in planar MM structures can have Q factors several orders higher. Below we present the results demonstrating the formation of resonances with similar high-Q factors in ordinary DR arrays.

## 3. Formation of Resonances with Fano-Type Specifics in Finite DR Arrays

**Figure 2:**
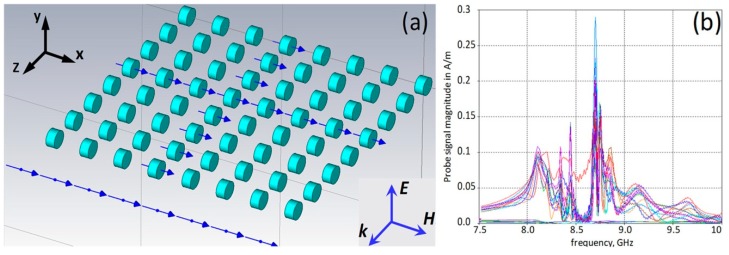
(**a**) DR array infinite due to periodic boundary conditions (PBC) in E-field direction at plane wave incidence along z-axis (arrows show H-field probe locations); and (**b**) spectra of probe signals. DR permittivity is 37, diameter = 6 mm, height = 3 mm.

Unusual resonance responses in DR arrays have been first detected by our group at the investigation of 3D MM structures (Figure 2a) composed of cylindrical DRs [16,36]. The DR dimensions were much smaller than the wavelength in air at the frequency of the magnetic Mie resonance; therefore, the structures were expected to represent homogenized MM media. Although the spectra of magnetic field probe signals in DRs demonstrated splitting, the formation of a stop-band typical for a homogenized MM medium with Lorentz-type response was clearly observed at frequencies between 8.1 GHz and 8.65 GHz (Figure 2b). According to the Lorentz-Mie theory, the stop bands in MMs should appear due to the change of the phase of resonance oscillations at *f = f_res_* by 180°, that, in turn, should reverse the sign of the medium effective parameter (permeability at the magnetic resonance) to a negative one [37]. This negativity should be maintained up to the magnetic plasma frequency *f = f_mp_* thus causing the stop-band at frequencies *f_res_ < f < f_mp_*. In frames of this approach no other resonances above *f_mp_*, *i.e.*, near the upper edge of the stop-band were expected in MMs.

Figure 2b, instead, demonstrates the appearance above the stop-band of a family of strong, narrow-band, very high-Q resonances, which, in contrary to the resonances observed below the stop-band at about 8.1 GHz, are accompanied by transmission peaks just as that at EIT studies in planar MMs [4,27,30,31]. Another intriguing feature of the resonances observed above the stop-band was their coherence in all DRs that can be an indication of a superluminal phase velocity of waves passing through DR chains positioned along the wave k-vector. This feature is characteristic for waves with a very low group velocity that prompts to consider passing waves at the respective resonance frequency as trapped, frozen, or slow, *i.e.*, characteristic for EIT.

The results of our recent studies of high-Q resonances in all-dielectric MMs have shown that these resonances apparently represent one of two states, in which Lorentz-Mie resonances can emerge in periodic resonance structures. This conclusion can be illustrated with the simulation results for dielectric rod arrangements formed by stacking planar rod arrays (PRAs) along the wave propagation direction. Each planar array of infinite rods was modeled by a unit cell with periodic boundary conditions (PBC) in two directions normal to *k*-vector. A piece of rod was placed in the center of such unit cell along the wave H-field direction. The stacks of planar layers were modeled by linear arrays of such unit cells.

**Figure 3:**
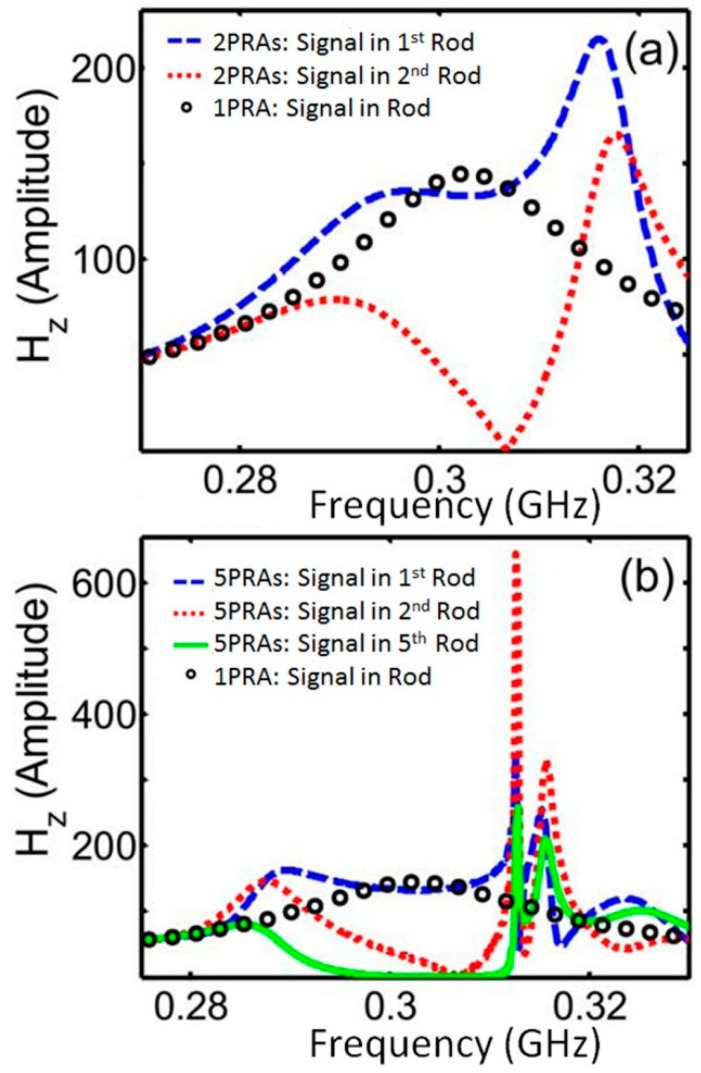
H-field probe spectra versus normalized frequency (*a*/*λ*) for a single PRA and arrays of (**a**) 2 and (**b**) 5 PRAs. Rod permittivity is 37.

As seen from Figure 3a, while a single PRA demonstrates characteristic for Lorentz-Mie resonances bell-type line-shape, addition of even one more PRA causes appearance of two distinct peaks in the probe signal spectra for the second PRA, while the response in the first PRA becomes stretched between two peaks positioned close to the frequencies of split resonances in the second PRA. Adding more stacked PRAs did not lead to additional splitting. Instead, the spectra of H-field probes placed in the rod centers demonstrated enhancement of asymmetrically shaped peaks above the gradually forming stop band (Figure 3b).

**Figure 4:**
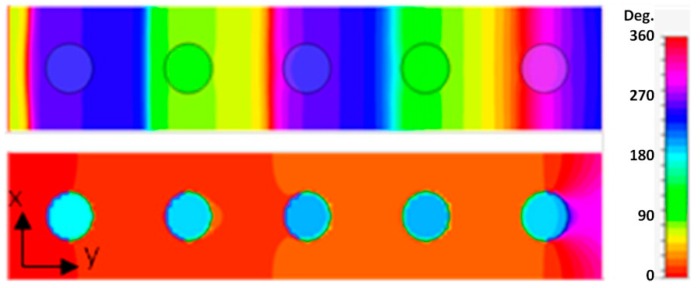
H-field phase patterns in cross-section of rods for 5 PRA array at frequencies: 0.29 (upper row) and 0.312 (lower row).

**Figure 5:**
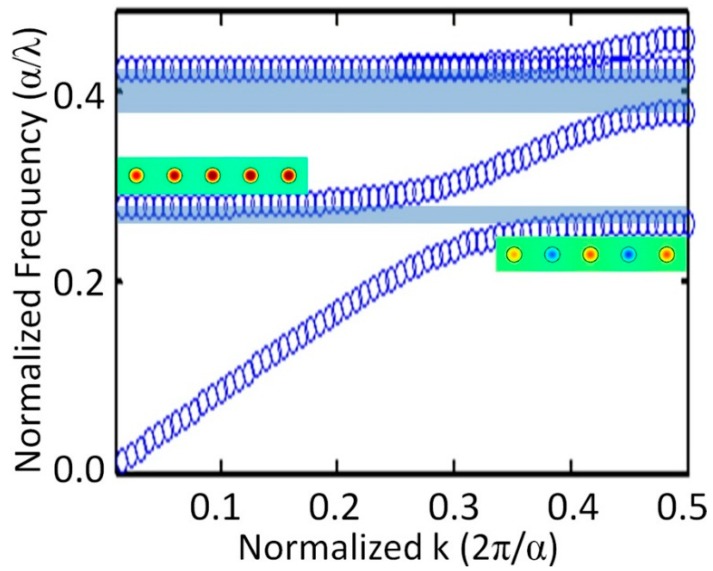
The Г-X part of 4 lower branches in the band diagram of DR array at the rod dielectric constant of 37. The bandgaps between transmission bands are colored grey. The inserts present resonance mode patterns observed at the upper edge of the 1st (odd mode) and lower edge of the 2nd (even mode) transmission bands. For the 2nd branch ∂*ω*/∂*k* → 0 at small *k*, so that waves have slow group velocity and infinite phase velocity apparently causing coherent resonance oscillations in the array.

Resonances observed near two opposite edges of the stop band formed different modes along the direction of wave propagation (Figure 4). For the lower frequency odd mode, the phases of resonance oscillations in neighboring resonators along the wave propagation direction were shifted by 180°, while at the higher frequency even mode a coherent response was observed in all resonators of the array. The difference in mode frequencies is not surprising, when one considers the action of repulsive or attracting forces between magnetic moments of neighboring rods for two modes. In fact, the formation of odd and even modes in multi-resonator systems due to interaction between the resonance moments could be considered as a pretty general phenomenon comparable to the formation of bonding and anti-bonding modes in molecules [38,39,40]. The performed simulations of photonic energy band diagrams for infinite rod arrays have shown that odd and even modes always appear near the closest edges of neighboring transmission bands (Figure 5) and thus could be considered as two respective transmission modes, while the stop-band dividing the two modes (that originated from the point with zero probe signal between two resonances in Figure 3a) could be seen as resulting from distractive interference between the incident and scattered by resonators waves.

It should be noted that photonic energy band diagrams simulated for infinite rod arrays have shown that even modes are always observed at frequencies corresponding to close-to-zero values of *k*-vector and to ∂*ω*/∂*k* → 0 at the edge of the second transmission band. Such independence on *k*-vector is usually characteristic for local resonances weakly coupled to incident waves, *i.e.*, for dark and slow transmission modes. These properties of even modes are in agreement with coherence of resonances and EIT-like high transmission, which should be free from scattering loss, if resonances are weakly coupled to incident waves.

The described results are in favor of Fano-type specifics of unusual high-Q resonances observed in DR arrays above the stop-band. It should be noted here that our earlier studies of Mie scattering by high-index dielectric resonators [3] have confirmed that the Lorentz-Mie coefficients in the Mie problem can be expressed as infinite series of Fano functions describing interference between the background radiation caused by incident waves and the narrow-spectrum Mie scattering modes. Apparently, similar interference should take place between the continuum of waves belonging to transmission bands of rod arrays and local Mie resonances in rods. However, to be cautious, we prefer to name the observed high-Q resonances in DR arrays as the FR-like resonances and not as FR. 

## 4. The Role of Fabry-Perot Resonances

**Figure 6:**
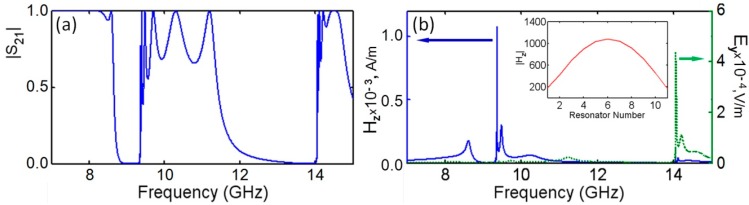
(**a**) Transmission spectra of finite DR array showing fringes near the edges of the 2nd and 3rd transmission bands; (**b**) signal spectra of H- and E-field probes.

**Figure 7:**
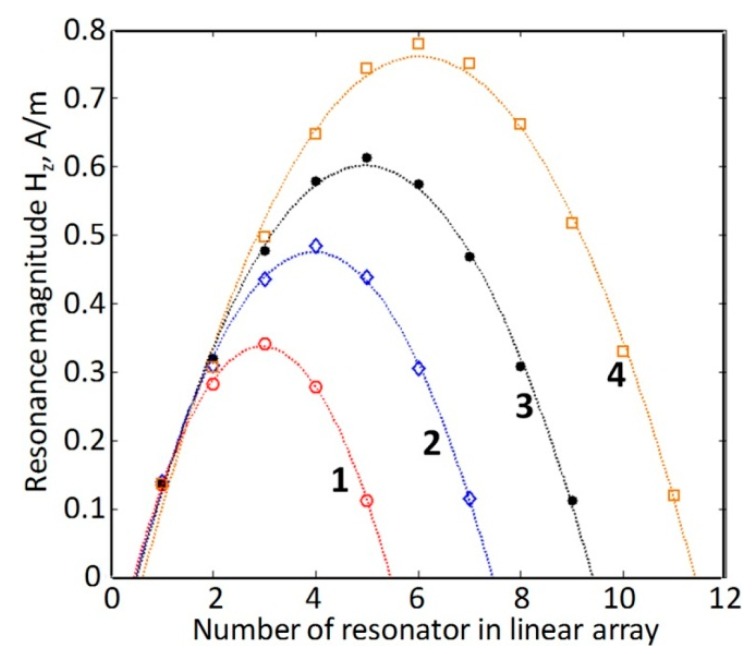
Signal magnitudes in probes placed inside the rods in linear arrays of unit cells used to model 3D DR arrays composed of 5 (curve 1), 7 (curve 2), 9 (curve 3) and 11 (curve 4) planar (2D) DR arrays.

The fact that the FR-like resonances observed in DR arrays above the transmission gap are accompanied by full resonance transmission and, thus, can be considered as related to transmission resonances, which are responsible for fringes in transmission spectra of PhCs [41], let us suggest that waves participating in the above transmission contribute to formation of the FR-like resonances. This means that in addition to expected interference with local resonances leading to Fano shape of combined array response, such waves can, possibly, pump local resonances causing their strengthening. The characteristic features of transmission resonances allow for relating them to the Fabry-Perot type of resonances, which can be considered as oscillations of standing waves formed by oppositely propagating along the direction of wave incidence Bloch eigenmodes, which experience reflections from array ends. Similar to the pumping phenomenon in lasers, it is possible to expect energy transfer from Fabry-Perot oscillations to local Mie resonances, since both operate at the same resonance frequencies. The Fabry-Perot resonances in PhCs are well studied [42] and they could be expected in periodic DR arrays. The character of frequency shifts in series of such resonances within the second transmission band of the rod arrays (Figure 6) perfectly corresponds to the case of Fabry-Perot resonances in PhCs that allows to consider the left, most sharp resonance in this series as the lowest order Fabry-Perot resonance corresponding to the formation of a standing wave, half-wavelength of which is equal to the length of the sample [43,44] Figure 7, which presents the distributions of magnitudes for local resonances along the array build by using the probe signal data for arrays of different DR sets, confirms such consideration. The fact that strongest FR-like responses are seen exactly in the center of arrays, where magnitudes of Fabry-Perot oscillations for first fringes should reach highest values is in favor of the suggestion about pumping local resonances by the Fabry-Perot oscillations. It seems also justified to relate the EIT-like transmission to the “transmission resonances”. As it was recently shown, transmission resonances in PhC are typically accompanied by slow waves, *i.e.*, by the same phenomenon as the EIT-like peaks in planar MMs [44,45].

## 5. Designing and Prototyping Sensing Devices with Implemented Compact DR Arrays

**Figure 8:**
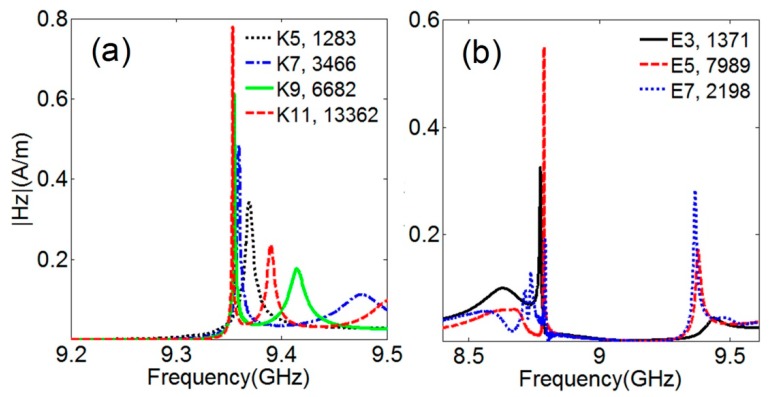
Fano resonances in (**a**) infinite in E-field direction arrays composed of the number of PRAs shown in insert; and (**b**) arrays composed of five PRAs with the widths restricted in E-field direction by the number of DR lines shown in insert. Q factors are shown by the second numbers in insets. DR relative permittivity is 37.

From the presented above data demonstrating the formation of high-Q resonances above the stop-bands of 3D DR arrays (Figure 2 and Figure 3) it follows that observation of such resonances requires composing respective metamaterial arrays of, at least, five or more planar 2D arrays stacked along the *k*-direction. This means that the array application in sensors a relatively long extension of array lattices along the wave propagation direction should be provided. The signal spectra obtained at H-field probe monitoring of local resonances in the center cells of linear arrays used to model 3D DR arrays extended in *k*-direction (Figure 8a) illustrate the importance of the above demands. As seen in the figure, the Q factors exceeding 10,000 can be obtained for local resonances only at the lengths of arrays along the wave propagation direction equal to 10 cells or more. The above discussion about the role of Fabry-Perot resonances in excitation of local FR-like resonances allows for relating these conditions to those necessary for obtaining strong Fabry-Perot resonances. Less clear is the effect of array extension along two other directions normal to the k-vector (along one direction in case of infinite rods). In particular, it was found that restricting the array dimension in E-field direction by several lattice parameters (6, 4 or 2) can cause decreasing of the Q factor for resonances above the stop band. On the other hand, the decay of these resonances appears accompanied by enhanced the FR-like resonances below the stop-band with the Q factor approaching 2200 at six periods and 8000 at four periods of the array width along the E-field direction (Figure 8b). This result indicates that relatively compact arrays still can demonstrate high enough Q factors of resonance response. 

Another challenge for practical sensing is seen in realizing proper excitation of DR arrays comparable with plane wave excitation used in simulations. A conventional practice at employment of microwave resonators involves using standard microwave waveguides as feeding system. Waveguides, however, are relatively bulky; therefore, feeding microwave sensors by miniaturized microstrip lines or coplanar waveguides becomes more popular [46]. To a pity, the width of the wave path in microstrip lines and coplanar waveguides, however, is relatively narrow that can create problems for resonator excitation. In addition, in difference from waveguides, narrow microstrips (or slots) could deteriorate the mirror effect, which is useful for virtual increasing the effective array dimension in E-field direction.

**Figure 9:**
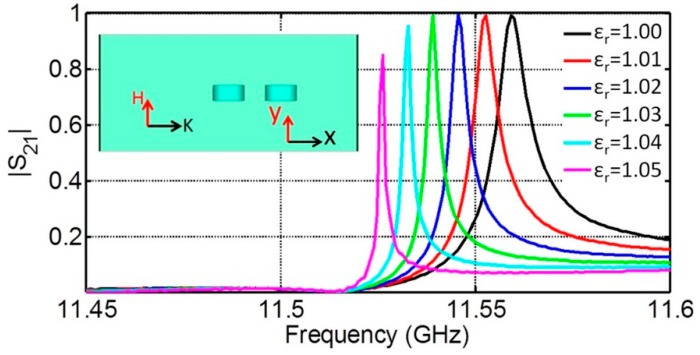
Transmission spectra of the waveguide with two inserted DRs filled by the media with the relative permittivities of 1.0; 1.01; 1.02; 1.03; 1.04 and 1.05, respectively. DR relative permittivity is 23.

A possibility of FR-based sensing at waveguide feeding is exemplified by Figure 9, insert of which shows a piece of a waveguide with inserted simplest array of two DRs with the relative permittivity of 23. Taking into account a mirror-like effect of waveguide walls, this arrangement represents an infinite in H- and E-field directions DR array of two planar components under TEM wave incidence, which, according to the previously presented results, should exhibit resonances with relatively high-Q factors. The array responses at FR frequencies were sampled by simulating the S_21_ spectra (Figure 9). To test the sensitivity of these responses to ambient substances, the inner space of the waveguide was “filled” with various background media having close to 1 relative permittivity values that were varied by 0.01. As seen from Figure 9, the obtained transmission spectra had asymmetric line shapes corresponding to expectations for FRs with characteristic transmission at the resonance frequencies. The changes of background permittivity caused well distinguished shifts of FR frequencies by about 0.0065 GHz.

**Figure 10:**
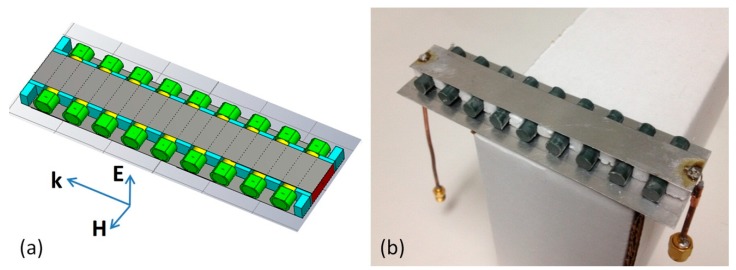
(**a**) Model of a microstrip line-fed sensor with rod array under the strip. Small amount of controlled substance is modeled by caps at the rod ends; (**b**) prototype of the microstrip line-fed sensor with rod array under the strip.

The design of array based sensor employing microstrip line feeding is exemplified by Figure 10, where the 24 mm-long dielectric resonators with the relative permittivity equal to 37.2 are represented by yellow cylinders placed between the 14 mm by 114 mm microstrip and the 34 mm by 114 mm ground plane and separated by blue colored pieces of foam (the separation distance is 6 mm). The microstrip line parameters were chosen to provide full transmission at FR frequency. It was expected that excitation of Fabry-Perot resonances in the linear array in combination with a mirror effect will cause the formation of strong high-Q Fano-type resonances, which could be detected as either peaks of EIT-like transmission or reflection dips in the S-parameter spectra by a network analyzer. Figure 10b presents a photograph of the fabricated sensor prototype with the same dimensions as those of the simulated model, where DRs were represented by ceramic rods placed in the foam niches underneath the microstrip line. The ends of the rods could be either kept in free contact with ambient or covered by caps of polymer with close to ambient permittivity to ensure achieving the desired sensing efficiency.

**Figure 11:**
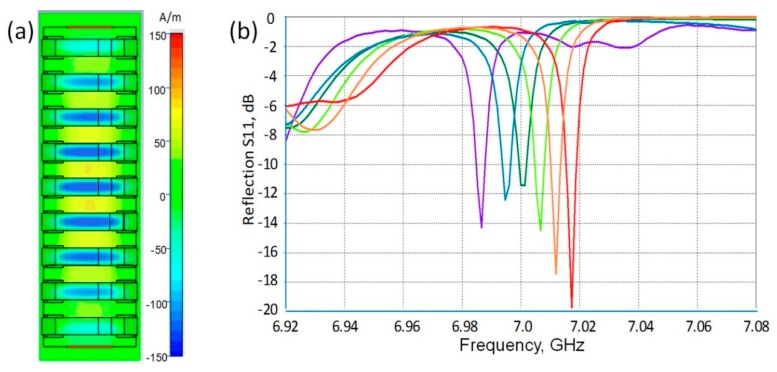
(**a**) Simulated field pattern in the cross-section of the sensor presented in Figure 10 confirming the formation of sin-phase Fano-type resonances in the DR array; (**b**) shifts of resonance related S_11_ dips (from red to violet) at changing the analyte relative permittivity from 1 to 3 by equal steps.

Simulation results presented in Figure 11 demonstrate the performance of the proposed sensor design. The field pattern in the array cross-section normal to the E-field direction (Figure 11a) displays coherent resonances in rods controlled by the half-wavelength Fabry-Perot mode (standing wave). The S_11_ spectra (Figure 11b) demonstrate meaningful shifts of resonance related dips in dependence on the analyte permittivity although the amount of the analyte was chosen intentionally to be very small and was modeled by just 125 microns thick caps at the rod ends. Simulation results promise an increase of FR Q factor up to 1500 at increasing the rod number in the array up to 15. Although the described design has not yet been optimized, the obtained data look well competitive on the sensor market and confirm the potential of the new approach for developing resonance sensors that will surpass existing devices.

**Figure 12:**
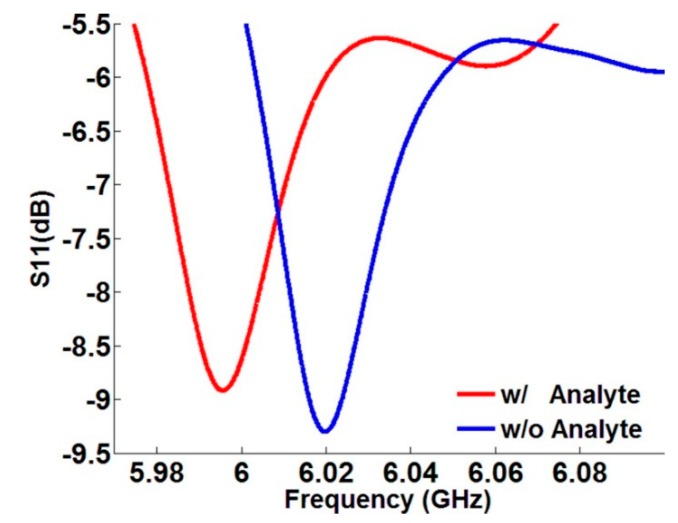
Shifting of the resonance dip in the S_11_ spectrum of the sensor prototype, when polymer phantoms of analyte with the relative permittivity of about 2 were inserted in air gaps between resonators.

The results of preliminary experiments with the prototype are shown in Figure 12, where the S_11_ spectrum demonstrates similar behavior as it does in simulations. When polymer phantoms of analyte were applied, the dip in the spectrum shifted to the lower frequency. Because of the fabrication tolerance, the prototype device worked at lower frequencies compared to those in the simulation model and the dips were not sufficiently sharp. Prototyping based on a variety of models and experiments are currently in progress.

## 6. Conclusions

Conducted studies have demonstrated that MM arrays of DRs are capable of responding to microwave irradiation by the formation of resonances with extremely high-Q factor up to 15,000. These resonances are apparently related to the even or anti-bonding resonance mode, which represents one of two energy states for magnetic Mie resonances in the arrays. Thus, the characteristic frequency of the above mode corresponds to the bottom of the second transmission band in the energy diagram of the DR array. We infer that the high-Q factor, specific Fano-type shape, and remarkable strength of the observed resonances are defined by the interaction of local DRs with Fabry-Perot oscillations of slow background waves. The latter oscillations reveal themselves in accompanying transmission resonances, at which the distribution of local resonance magnitudes along the array corresponds to the shape of Fabry-Perot standing waves. Such mechanism opens up an opportunity for controlling the Q factor and the strength response at high-Q resonances by changing DR arrangement in arrays. Performed modeling and prototyping confirmed the possibility to design microwave sensors operating due to Fano-type resonances excited in compact DR arrays. The conducted studies create a conceptual basis for future designing of a new class of efficient sensors for a wide range of applications in microwave and mm-wave ranges. It is also worth noting that prototyping and characterization of DR-based MMs for incorporating in microwave range sensors is more feasible and cheaper than similar procedures with MMs for optical range.
